# The Outcomes of Intracytoplasmic Sperm Injection and Laser Assisted Hatching in Women Undergoing *In Vitro* Fertilization Are Affected by The Cause of Infertility

**DOI:** 10.22074/ijfs.2015.4206

**Published:** 2015-04-21

**Authors:** Hsin-Fen Lu, Fu-Shiang Peng, Shee-Uan Chen, Bao-Chu Chiu, Szu-Hsing Yeh, Sheng-Mou Hsiao

**Affiliations:** 1 Division of Reproductive Endocrinology and Infertility, Department of Obstetrics and Gynecology, Far Eastern Memorial Hospital, New Taipei, Taiwan; 2 Department of Obstetrics and Gynecology, National Taiwan University Hospital, Taipei, Taiwan

**Keywords:** Assisted Reproduction Technology, *In Vitro* Fertilization, Intracytoplasmic Sperm Injection

## Abstract

**Background:**

We sought to determine the association between factors that affected clini- cal pregnancy and live birth rates in patients who underwent *in vitro* fertilization (IVF) and received intracytoplasmic sperm injection (ICSI) and/or laser assisted hatching (LAH), or neither.

**Materials and Methods:**

In this retrospective cohort study, the records of women who underwent IVF with or without ICSI and/or LAH at the Far Eastern Memorial Hospital, Taipei, Taiwan between January 2007 and December 2010 were reviewed. We divided patients into four groups: 1. those that did not receive ICSI or LAH, 2. those that received ICSI only, 3. those that received LAH only and 4. those that received both ICSI and LAH. Univariate and multivariate analyses were performed to determine factors associated with clinical pregnancy rate and live birth rate in each group.

**Results:**

A total of 375 women were included in the analysis. Oocyte number (OR=1.07) affected the live birth rate in patients that did not receive either ICSI or LAH. Mater- nal age (OR=0.89) and embryo transfer (ET) number (OR=1.59) affected the rate in those that received ICSI only. Female infertility factors other than tubal affected the rate (OR=5.92) in patients that received both ICSI and LAH. No factors were found to affect the live birth rate in patients that received LAH only.

**Conclusion:**

Oocyte number, maternal age and ET number and female infertility fac- tors other than tubal affected the live birth rate in patients that did not receive ICSI or LAH, those that received ICSI only, and those that received both ICSI and LAH, respectively. No factors affected the live birth rate in patients that received LAH only. These data might assist in advising patients on the appropriateness of ICSI and LAH after failed IVF.

## Introduction

Since the introduction of assisted reproductive technology (ART), in vitro fertilization (IVF) has enabled countless couples to achieve pregnancy. However, failure to conceive after multiple attempts with different methods imparts a significant emotional and financial burden on patients (1-4). It has been estimated that up to 85% of embryos do not implant (5, 6). Many attempts have been made to identify factors that can predict the success of IVF and it is generally accepted that female age, duration of subfertility, baseline follicle stimulating hormone (FSH) levels, and number of oocytes are predictors of pregnancy after IVF (7, 8). In our prior study, we have identified that the number of embryos transferred, the presence of ovarian hyperstimulation syndrome, female infertility factors other than tubal factors, and embryo quality were correlated with the failure to achieve birth emphasizing a successful singleton at term (BESST) (i.e., the singleton, term gestation and live birth) (9, 10). Other studies have shown that IVF success is associated with the diagnosis after an infertility workup, the number of previous unsuccessful IVF attempts, and a prior successful pregnancy; however, no truly useful model for predicting the success of IVF exists (11).

Depending on the reasons for infertility in a particular couple, numerous techniques such as intracytoplasmic sperm injection (ICSI) and assisted hatching (AH) have been developed to increase the probability of pregnancy and a live birth (5, 12, 13). ICSI is typically used for male factor infertility and in cases where eggs cannot easily be penetrated by sperm. Despite the concern for genetic abnormalities, it is a proven technique for achieving successful pregnancy and live birth (14, 15). It is well known that a proportion of euploid embryos fail to implant because of hatching difficulties (15) and AH involves artificial disruption of the zona pellucida with the intent of increasing implantation potential (16). Many methods have been developed to disrupt the zona pellucida and laser AH (LAH) has been found to be more effective in some subgroups of patients (12, 17). However, a recent analysis by Myers et al. (18) has concluded that there is relatively little high-quality evidence to support the choice of specific interventions.

The purpose of this study was to determine the association of factors that affected the clinical pregnancy and live birth rates in patients that underwent IVF who received both ICSI and LAH, neither ICSI or LAH, or only ICSI or LAH.

## Materials and Methods

In this retrospective cohort study the outcomes of women who underwent IVF with or without ICSI at the Far Eastern Memorial Hospital, Taipei, Taiwan between January 2007 and December 2010 were reviewed. Cases in which estradiol levels exceeded 50 pg/mL on the second day of the menstrual cycle were excluded. The study was approved by the Research Ethics Review Committee of the Far Eastern Memorial Hospital. Due to the retrospective nature of the study the requirement for informed consent was waived.

Causes of reduced female fertility included tubal causes, endometriosis, anovulation, polycystic ovary syndrome (PCOS), decreased ovarian reserve, uterine disorders, age >35 years (advanced maternal age) and unidentified reasons. Females might have had one or multiple factors. Male causes of infertility were decreased sperm concentration (<2×10^7^/ml), decreased sperm motility (<50%) and azoospermia. Patients with one or more of the following criteria underwent ICSI: 1. fertilization rate below 50% in a prior IVF attempt and 2. male factor infertility. In cases of azoospermia, sperm for ICSI was obtained by microsurgical epididymis sperm aspiration (MES) or testicular sperm extraction (TESE). Patients with one or more of the following criteria underwent LAH: 1. zona pellucida >15 μm, 2. maternal age over 38 years and 3. at least three failed IVF attempts.

LAH was performed in a standard manner. Briefly, a 1.48 μm infrared diode laser (OCTAX Laser Shot™ System, Medical Technology Vertriebs-GmbH, Germany) in a computer-controlled non-contact mode was used. After positioning the embryo, the laser was focused at the equatorial level of the zona pellucida. A pulse length of 2.8 ms was used and the LAH procedure was performed until 25% of the zona pellucida was drilled.

The method of ovulation induction used in the study center was previously published (9). In brief, gonadotropin-releasing hormone agonist (Supremon, Aventis Pharma Deutschland, Frankfurt, Germany) was administered from the third day of the menstrual cycle via nasal spray, daily, in 4 doses of 200 μg. FSH (Gona-F, Serono, Geneva, Switzerland), 150-225 IU, was administered daily from the fifth day of the menstrual cycle via subcutaneous injection into the abdomen. Luteinizing hormone (LH) and estradiol levels were measured from the seventh day of the cycle, and transvaginal ultrasonography was performed every two days in order to adjust dosages until complete follicular growth was achieved. When appropriate follicular growth was detected, 10000 IU of human chorionic gonadotropin (hCG, Pregnyl, NV Organon, Oss, The Netherlands) was injected and oocyte retrieval was performed 35 hours later. At four hours after oocyte retrieval, IVF was carried out, with or without ICSI. Two to five days later, embryos at the 4-cell to blastocyst stage were transferred; the remainder were frozen and stored in liquid nitrogen.

Pregnancy was defined as a βhCG level greater than 50 mIU/mL 14 days after day 2 embryo transfer (ET). Clinical pregnancy was defined by the ultrasound observation of fetal cardiac activity. We defined live birth as the birth of a newborn, irrespective of the duration of gestation that exhibited any signs of life.

For analysis, patients were divided into four groups: 1. those that did not receive either ICSI or LAH, 2. those that received ICSI only, 3. those that received LAH only and 4. those that received both ICSI and LAH.

### Statistical analysis

For comparability among the four groups we used one-way analysis of variance (ANOVA) for normally distributed continuous variables and the chi-square test for categorical variables. If the data was nonnormally distributed, Kruskal-Wallis tests were used to determine the difference among the four groups. When significance among group differences were apparent, multiple comparisons of means were performed using the Bonferroni procedure with type-I error adjustment. Parametric variables were represented as mean and standard deviation (SD) and categorical data were represented by number (n) and percentage (%). Nonparametric variables were represented as median (inter-quartile range). Univariate logistic regression analysis was performed to analyze the odds ratio (OR) of significant factors associated with successful pregnancy and live birth. Variables having a p value <0.05 in the univariate analysis were selected and evaluated by multivariate logistic regression models with the conditional forward selection method. All statistic assessments were two-sided and evaluated at the 0.05 level of significance. Statistic analyses were performed using SPSS 15.0 statistics software (SPSS Inc., Chicago, IL, USA).

## Results

After applying the inclusion and exclusion criteria, a total of 375 women who underwent IVF between January 2007 and December 2010 were included in the analysis. The mean age of patients was 34.1±4.7 years, and the mean age of their partners was 37.3 ± 5.4 years. In total, 121 patients (32.2%) did not receive either ICSI or LAH, 176 patients (46.9%) received ICSI only, 22 patients (5.9%) had LAH only, and 56 patients (14.9%) underwent both ICSI and LAH. The demographic and clinical characteristics of the patients are shown in table 1. There were signiﬁcant differences in the age of partners, age of the patients, duration of infertility, the reason for infertility (tubal factor, other female factors, and male factor), number of previous IVF courses, oocyte number, and embryo number among the four groups (p<0.05).

In total, 179 (47.7%) women became pregnant. Of these, 126 (33.6%) had subsequent live births. The results of the univariate and multivariate analyses for factors that affected clinical pregnancy rate are shown in tables 2 and 3, respectively. Multivariate logistic regression indicated that only advanced maternal age affected clinical pregnancy rate in those that did not receive either ICSI or LAH (OR=0.87, 95% CI: 0.78 to 0.96, p=0.005). In patients that received ICSI only, advanced maternal age (OR=0.93, 95% CI: 0.86 to 0.99, p=0.044), female factors other than tubal (OR=3.37, 95% CI: 1.26 to 19.05, p=0.016) and embryo number (OR: 1.10, 95% CI: 1.03 to 1.18, p=0.007) affected the clinical pregnancy rate. In patients that received LAH only, only embryo number (OR=3.26, 95% CI: 1.24 to 8.57, p=0.017) affected the clinical pregnancy rate. In those that received both ICSI and LAH, only male factor (OR=0.32, 95% CI: 0.11 to 0.97, p=0.044) affected the clinical pregnancy rate.

The results of univariate and multivariate analyses of factors influencing the live birth rate are shown in tables 4 and 5, respectively. Multivariate logistic regression analysis indicated that oocyte number (OR=1.07, 95% CI: 1.01 to 1.13, p=0.031) affected the live birth rate in patients that did not receive either ICSI or LAH. In patients that received ICSI only, advanced maternal age (OR=0.89, 95% CI: 0.82 to 0.96, p=0.004) and ET number (OR=1.59, 95% CI:1.05 to 2.418, p=0.027) affected the live birth rate. In patients that received both ICSI and LAH, female factors other than tubal affected the live birth rate (OR=5.92, 95% CI:1.14 to 30.73, p=0.016). No factors were found to affect the live birth rate in patients that received LAH only.

**Table 1:** Patient demographic clinical characteristics (n=375)


	Total (n=375)	Neither ICSI or LAH (n=121)	ICSI (n=176)	LAH (n=22)	Both ICSI and LAH (n=56)	P value

**Age of partner (Y)^1^**	37.30±5.40	36.54±5.34	37.10±5.47	37.48±3.94	39.49±5.36^†‡^	0.007*
**Age of patient (Y)^1^**	34.08±4.66	33.43±4.08	33.43±4.77	35.81±4.46	36.83±4.50^†‡^	<0.001*
**Duration of infertility (Y)^2^**	4 (2, 6)	4(2, 6)	3(2, 5.5)	4(2, 7)	6(4, 10) ^†‡^	<0.001*
**Cause of infertility^3^**						
**Tubal factor**	83(22.1)	46 (38.0)	25(14.2)^†^	5(22.7)	7(12.5)^†^	<0.001*
**Female factors other thantubal (e.g., endometriosis,PCOS, uterine disorders)**	96(25.6)	35 (28.9)	33(18.8)	9(40.9)^‡^	19(33.9)	0.020*
**Male factor**	136 (36.3)	15 (12.1)	97(55.1)^†^	1(4.5)^‡^	23(41.1)^†§^	<0.001*
**Combined femaleand male factors**	23(6.1)	5(4.1)	13(7.4)	2(9.1)	3(5.4)	0.634
**Previous IVF course^2^**	0 (0, 1)	0(0, 0)	0(0, 0)	0(0, 0)	0(0, 1.5)^†‡^	0.021*
**Oocyte number^2^**	9 (4, 14)	8(4, 13)	10(5.5, 15)	6.5 (2, 14)^‡^	6(4, 12)^‡^	0.005*
**Embryo number^2^**	5 (3, 9)	4(2, 9)	6(3, 10)	4(1, 7)	4(3, 6)	0.045*
**ET number^2^**	3 (2, 4)	3(2, 4)	3(3, 4)	3(1, 4)	3(2, 4)	0.110
**Reason for ICSI**						
**Maternal age >35 years**	138 (40.2)	0	94(64.3)	0	44(82.6)	NA
**Fertilization rate <50%in prior IVF attempt**	61(17.8)	0	39(25.8)	0	22(42.3)	NA
**Male factor**	121 (35.3)	0	98(64.9)	0	23(44.2)	NA
**Reason for LAH**						
**Maternal age >38 years**	63(16.9)	0	0	18 (81.8)	45(80.4)	NA
**Zona pellucida >15 µm**	23(6.2)	0	0	8(36.4)	15(26.8)	NA


ET; Embryo transfer, ICSI; Intracytoplasmic sperm injection, IVF; In vitro fertilization, LAH; Laser assisted hatching, PCOS; Polycystic ovary syndrome, *; Indicates a significant difference,p<0.05, †; Indicates a statistically significant difference between the indicated group and the group that did not receive ICSI or LAH group, ‡; Indicates a statistically significant difference between the indicated group and the ICSI group, §; Indicates a statistically significant difference between the LAH and both ICSI and LAH groups, p values are based on 1; ANOVA, 2; Kruskal-Wallis test and 3; Chi-square test. Data are presented as mean±standard deviation, number (percentage), or median (interquartile range). Pair-wise multiple comparisons between groups were determined using Bonferroni’s test with α=0.008 adjustment.

**Table 2:** Results of univariate analysis for factors that affected clinical pregnancy rates in the four groups


	Neither ICSI or LAH OR (95% CI)	ICSI OR (95% CI)	LAH OR (95% CI)	Both ICSI and LAH OR (95% CI)

**Age of partner (Y)**	0.90(0.84- 0.97)*	0.98(0.93-1.04)	0.83(0.65-1.07)	0.95(0.86-1.05)
**Age of female (Y)**	0.87(0.76- 0.96)*	0.89(0.83-0.95)*	0.94(0.77-1.15)	0.97(0.86-1.09)
**Duration of infertility (Y)**	0.95(0.83-1.07)	0.97(0.87-1.08)	0.89(0.67-1.18)	1.00(0.87-1.14)
**Tubal factor (no vs. yes)**	1.58(0.74-3.34)	1.56(0.67-3.64)	0.59(0.08-4.50)	0.45(0.08-2.56)
**Female factors otherthan tubal (no vs. yes)**	0.95(0.43-2.09)	4.20(1.63-10.78)*	7.88(1.01-56.12)*	3.29(0.98-11.03)
**Male factor (no vs. yes)**	1.55(0.52-4.59)	0.64(0.35-1.18)	-	0.32(0.11-0.97)*
**Multiple factors (no vs. yes)**	1.98(0.32-12.30)	0.30(0.09-1.02)	-	1.66(0.14-19.39)
**Previous IVF course**	1.05(0.87-1.28)	0.56(0.35-0.97)*	-	0.89(0.65-1.22)
**Oocyte number**	1.09(1.02-1.15)*	1.07(1.02-1.11)*	1.08(0.93-1.26)	0.98(0.90-1.07)
**Embryo number**	1.09(1.01-1.18)*	1.14(1.06-1.22)*	1.13(0.93-1.38)	0.95(0.83-1.09)
**ET number**	1.55(1.05- 2.27)*	1.64(1.19-2.27)*	3.26(1.24-8.57)*	1.36(0.79-2.33)


CI; Confidence interval, ET; Embryo transfer, ICSI; Intracytoplasmic sperm injection, IVF; In vitro fertilization, LAH; Laser assisted hatching, OR; Odds ratio and *; Significance: p<0.05.

**Table 3:** Results of multivariate analysis for factors that affected clinical pregnancy rates in the four groups


	Neither ICSI or LAH OR (95% CI)	ICSI OR (95% CI)		LAH OR (95% CI)	Both ICSI and LAH OR (95% CI)

**Age of female (Y)**	0.87 (0.78-0.96)*	0.93	(0.86-0.99)*	-	-
**Female factors other than**	-	3.37	(1.26-19.05)*	-	-
**tubal (no vs. yes)**					
**Male factor (no vs. yes)**	-	-		-	0.32 (0.11-0.97)*
**Embryo number**	-	1.10	(1.03-1.18)*	3.26 (1.24-8.57)*	-


CI; Confidence interval, ICSI; Intracytoplasmic sperm injection, IVF; In vitro fertilization, LAH; Laser assisted, OR; Odds ratio and *; Significance: p<0.05.

**Table 4:** Results of univariate analysis for factors that affected live birth rate in the four groups


	Neither ICSI or LAH OR (95% CI)	ICSI OR (95% CI)	LAH OR (95% CI)	Both ICSI and LAH OR (95% CI)

**Age of partner (Y)**	0.94(0.87-1.01)	0.96(0.90-1.03)	0.82(0.64-1.06)	0.92(0.82-1.03)
**Age of female (Y)**	0.91(0.83-1.01)	0.87(0.80-0.94)*	0.86(0.69-1.07)	0.89(0.78-1.02)
**Duration of infertility**	0.92(0.81-1.06)	0.94(0.83-1.07)	0.92(0.69-1.22)	0.87(0.74-1.03)
**Tubal factor (no vs. yes)**	0.73(0.35-1.55)	0.56(0.23-1.34)	1.05(0.14-8.02)	1.33(0.23-7.58)
**Female factors other thantubal (no vs. yes)**	1.57(0.68-3.61)	3.55(1.18-10.69)*	4.08(0.60-27.65)	7.23(1.46-35.84)*
**Male factor (no vs. yes)**	1.31(0.42-4.11)	0.57(0.29-1.12)	-	0.25(0.77-0.79)*
**Multiple factors (no vs. yes)**	0.95(0.15-5.91)	1.39(0.37-5.28)	0.67(0.04-12.27)	1.03(0.09-12.12)
**Previous IVF course**	0.93(0.74-1.16)	0.54(0.28-1.04)	-	0.88(0.62-1.25)
**Oocyte number**	1.07(1.01-1.13)*	1.06(1.02-1.11)*	1.05(0.91-1.21)	1.02(0.93-1.12)
**Embryo number**	1.06(0.98-1.14)	1.12(1.04-1.19)*	1.04(0.87-1.24)	1.01(0.88-1.14)
**ET number**	1.47(0.99-2.19)	1.84(1.23-2.73)*	1.97(0.87-4.42)	2.23(1.12-4.25)*


CI; Confidence interval, ET; Embryo transfer, ICSI; Intracytoplasmic sperm injection, IVF; In vitro fertilization, LAH; Laser assisted hatching, OR; Odds ratio and *; Significance: p<0.05.

**Table 5:** Results of multivariate analysis for factors that affected live birth rate in the four groups


	Neither ICSI or LAH OR (95% CI)	ICSI OR (95% CI)	LAH OR (95% CI)	Both ICSI and LAH OR (95% CI)

**Age of female (Y)**	-	0.89 (0.82-0.96)*	-	-
**Female factors other than**	-	-	-	5.92 (1.14-30.73)*
**tubal (no vs. yes)**				
**Oocyte number**	1.07 (1.01-1.13)*	-	-	-
**ET number**	-	1.59 (1.05-2.41)*	-	-


CI; Confidence interval, ET; Embryo transfer, ICSI; Intracytoplasmic sperm injection, IVF; In vitro fertilization, LAH; Laser assisted hatching, OR; Odds ratio and *; Significance p<0.05.

## Discussion

The results of this study showed that different factors affected the clinical pregnancy rate and live birth rate in patients who underwent IVF that received ICSI and LAH, neither ICSI or LAH, and ICSI or LAH only. In patients that received LAH only, only embryo number (OR=3.26, 95% CI: 1.24 to 8.57, p=0.017) affected the clinical pregnancy rate, and in those that received both ICSI and LAH only male factor (OR=0.32, 95% CI: 0.11 to 0.97, p=0.044) affected the clinical pregnancy rate. Furthermore, in patients that received only ICSI, advanced maternal age was associated with a decreased chance and ET number with an increased chance of live births; in patients that did not receive either ICSI or LAH oocyte number was associated with an increased chance of live birth.

Numerous attempts have been made to develop models that predict the success or failure of IVF, though few have been shown to be successful (11). While studies have clearly indicated that factors such as female age and baseline FSH levels are predictive of pregnancy after IVF, it remains difficult for physicians to advise patients on how to proceed after an IVF failure.

ICSI is commonly used to treat male factor infertility and in cases where the sperm cannot penetrate the egg. We have found that in patients that received only ICSI, only ET number was associated with an increased chance of having a live birth. Though ICSI has increased pregnancy and live birth rates in patients undergoing IVF, concerns remain regarding chromosomal abnormalities and some authors consider the procedure over used (19, 20). Tan et al. (21) compared the outcomes of IVF-ET (IVF) and ICSI in non-male infertility patients with low numbers of oocytes retrieved and reported that the rates of fertilization, normal fertilization, complete fertilization failure, cleavage, good embryo, implantation, and clinical pregnancy did not differ between the groups. The authors concluded that ICSI did not improve clinical outcomes in non-male infertility patients with a low number of oocytes retrieved. Hodes-Wertz et al. (22) studied the use of ICSI in couples who previously underwent ICSI at another institution and found that stringent criteria for ICSI did not compromise clinical outcomes and concluded that ICSI was over used.

We found that LAH alone was not associated with an increased live birth rate, but that the use of both ICSI and LAH was associated with an increased live birth rate in cases when female infertility factors other than tubal were not present. While AH and LAH are commonly used, a recent review by Hammadeh et al. (16) observed that routine use of AH was not appropriate as no evidence of a universal benefit existed and the procedure was not without potential risks. Ali et al. (17) reported that LAH was beneficial for women .36 years of age, embryos with a thin zona (.16 ƒÊm), and for those with repeated IVF failures. It was not beneficial for women .37 years of age or in cases in which the zona was .17 ƒÊm. Mansour et al. (23) reported a benefit of AH in patients with a poor prognosis such as those with two or more failed IVF cycles, poor embryo quality, and women >38 years of age. Petersen et al. (24) reported that for patients with repeated implantation failures, the implantation rate in those who received laser-thinned embryos was significantly higher (10.9%) than in those whose embryos were not laser-thinned (2.6%). This difference, however, was not seen in patients with only one previous implantation failure. A recent systematic review by Carney et al. (25) examined the effectiveness of AH and concluded that the increased chance of achieving a clinical pregnancy by AH only just reached statistical significance. The data did not support an increase in live birth rate. In our study, LAH did not increase the pregnancy or live birth rates. However, in table 3 LAH did increase the clinical pregnancy rate as related to embryo number. Combined with ICSI, in table 3 the results showed that in cases where infertility of the couple was caused by male factor, the clinical pregnancy rate increased significantly. Thus the use of assistance should be considered according to the special circumstances of each couple.

There are some limitations in this study that should be considered. First, this was a retrospective study, with a heterogeneous patient population. In addition, the numbers of patients in the subgroup that received only LAH was small.

## Conclusion

The results of this study indicate that the chance of a live birth in patients undergoing IVF and ICSI and/or LAH vary with the causes of infertility. Oocyte number, maternal age and ET number and female infertility factors other than tubal have affected the live birth rate in patients that did not receive ICSI or LAH, those that received ICSI only and those that received both ICSI and LAH, respectively. No factors affected the live birth rate in patients that received LAH only. These data might assist in advising patients on the appropriateness of ICSI and LAH after failed IVF.
